# Neuroprotective effects of the neurotrophic factors carbamoylated erythropoietin and insulin‐like growth factor‐1 in Alzheimer’s disease

**DOI:** 10.1002/alz70861_108019

**Published:** 2025-12-23

**Authors:** Morgan Jean Rothschadl, Monica Sathyanesan, Samuel S Newton

**Affiliations:** ^1^ University of South Dakota, Vermillion, SD USA

## Abstract

**Background:**

Cognitive functioning plays a significant role in determining an individual’s quality of life, with cognitive decline being a major symptom of Alzheimer’s disease (AD). Neurotrophic factors have been shown to reduce amyloid load and improve cognitive functioning in mouse models of AD; however, these studies have not examined whether combining two neurotrophic factors can enhance these benefits. Studies examining the neuroprotective properties of neurotrophic factors are also lacking. Here we investigate the neuroprotective potential of combining two neurotrophic factors, carbamoylated erythropoietin (CEPO) and insulin‐like growth factor‐1 (IGF‐1), in AD.

**Method:**

Male and female 5xFAD mice bred from both maternal and paternal origin were injected intraperitoneally with CEPO, IGF‐1, CEPO+IGF‐1, or vehicle starting at two months of age, before cognitive deficits are present, until nine months of age. Mouse behavior was analyzed using the open field and Morris Water Maze (MWM) tests at six and nine months of age to assess learning and memory. The brains were then removed to assess amyloid load and protein expression.

**Result:**

We found that male 5xFAD mice showed behavioral deficits in the MWM starting at six months of age, and that CEPO and IGF‐1 treatments, but not CEPO+IGF‐1, could protect against those deficits. At nine months, the neurotrophic factor‐treated male 5xFAD mice showed sustained improvement in the MWM test compared to the untreated 5xFAD mice.

The female 5xFAD mice did not show behavioral deficits in the MWM until nine months. At this age, the neurotrophic factor‐treated female 5xFAD mice did not show significantly different behavioral performance in the MWM test, indicating that these neurotrophic factors were ineffective at protecting against AD progression in female 5xFAD mice.

We also perform a correlative neuroanatomical analysis of amyloid load.

**Conclusion:**

The data demonstrate a clear sex‐specific difference in the neuroprotective effects of CEPO and IGF‐1 in protecting against cognitive decline, with these neurotrophic factors being effective in male but not female mice. Our data also suggest that combining two neurotrophic factors may not be more effective than their individual treatment; however, more studies looking at different dosing and timing regimens should be pursued in the future.

